# Meta-analysis of FTO expression on the clinicopathologic characteristics and prognosis of gastric cancer

**DOI:** 10.1097/MD.0000000000035714

**Published:** 2024-09-27

**Authors:** Ciba Zhu, Mingxu Da, Ziyao Wu, Jichun Ma, Chenglou Zhu, Xinqiao An, Dandan Ji, Chunling Xu

**Affiliations:** a The First Clinical Medicine College, Gansu University of Traditional Chinese Medicine, Lanzhou, China; b Department of Oncology Surgery, Gansu Provincial Hospital, Lanzhou, China; c The First Clinical Medicine College, Lanzhou University, Lanzhou, China; d Department of Hepatobiliary and Hernia Surgery, Wuwei Liangzhou Hospital, Wuwei, China.

**Keywords:** analysis, gastric cancer, fat mass and obesity, meta, related genes

## Abstract

**Background:**

Fat mass and obesity-related gene (FTO) is aberrantly expressed in various cancers including highly expressed in gastric cancer tissues. The aim of this meta-analysis was to explore the effect of FTO expression on clinicopathological and prognostic outcome of gastric cancer.

**Methods:**

China National Knowledge Infrastructure (CNK), Wanfang database, VIP database, Chinese biomedical literature database (CBM), PubMed, Web of Science, the Cochrane library and EMBASE database were searched to screen the literatures according to the inclusion criteria. The search time was the database establishment until May 2023. The two researchers independently searched and screened the literature, extracted pathological data, and conducted The Newcastle–Ottawa scale (NOS) quality evaluation. Analyze the correlation between FTO and pathological indicators of gastric cancer patients and the impact on prognosis, use and Stata 12.0, software for Meta-analysis.

**Results:**

A total of 1619 patients were studied in this study. The results of the Meta-analysis showed that higher expression levels of FTO were associated with TMN stage (OR = 1.83, 95% CI: 1.11–3.03, *P* = .019), liver metastases (OR = 3.73, 95% CI: 1.49–9.31, *P* = .005), vascular invasion (OR = 2.22, 95% CI: 1.36–3.61, *P* = .001), poorer overall survival (OS) (HR = 0.46, 95% CI: 0.34–0.58, *P* < .001) and recurrence-free survival (HR = 0.56, 95% CI: 0.40–0.73, *P* < .001) in gastric cancer patients. There was no significant relationship with the degree of differentiation (OR = 1.08, 95% CI: 0.49–2.35, *P* = .852), age (OR = 0.89, 95% CI: 0.71–1.11, *P* = .306), and gender (OR = 0.92, 95% CI: 0.74–1.14, *P* = .432).

**Conclusion:**

High expression of FTO was associated with risk of distant metastases and poor prognosis for patients with gastric cancer. FTO may be a potential prognostic biomarker for gastric cancer, but due to the limited number of literature, the above results need further research.

## 1. Introduction

Gastric cancer is one of the most common malignant tumors originating from the epithelial lining of the gastric mucosa in the digestive system. It predominantly presents as adenocarcinoma and ranks as the fourth leading cause of global mortality,^[1,2]^ accounting for approximately 8% of all cancers and 10% of cancer-related deaths. The incidence rate in males is approximately twice that in females,^[3]^ posing a significant threat to human health. Despite a declining trend in gastric cancer incidence globally in recent years, it remains highly prevalent in China, particularly in the eastern and northwestern regions, where it is among the areas with the highest incidence and mortality rates worldwide.^[4]^ Studies have shown that gastric cancer ranks fourth and third in terms of incidence and mortality rates, respectively, among malignant tumors in China.^[5,6]^ Gastric cancer often develops covertly, and due to the lack of early typical symptoms and screening indicators, 80% of patients are diagnosed at an advanced stage, resulting in a 5-year survival rate of less than 5%.^[7]^ Postoperative recurrence and metastasis are the primary reasons for the low 5-year survival rate in gastric cancer.^[8]^ Currently, clinical and pathological parameters are mainly used to predict prognosis and treatment response in gastric cancer patients.^[9]^ Therefore, it is crucial to identify potential biomarkers and therapeutic targets for the diagnosis and treatment of gastric cancer.

Fat mass and obesity-associated gene (FTO) is a protein-coding gene. FTO, also known as the obesity gene or ALKBH9,^[10]^ has been found to be aberrantly expressed in various cancers and associated with the risk of cancer development,^[11]^ such as breast cancer,^[12]^ thyroid cancer,^[13]^ endometrial cancer,^[14,15]^ pancreatic cancer,^[16,17]^ and others. Studies have indicated that FTO is highly expressed in gastric cancer and correlates with the pathological characteristics and prognosis of patients.^[18]^ However, due to the limited number of studies and divergent results, further research is needed. Therefore, this study aims to conduct a meta-analysis of relevant published literature to further explore the association between FTO expression and clinical pathology as well as its impact on prognosis in gastric cancer.

## 2. Materials and Methods

### 2.1. Search strategy

A comprehensive literature search was conducted using several databases, including China National Knowledge Infrastructure (CNKI), Wanfang database, VIP database, Chinese biomedical literature database (CBM), PubMed, Web of Science, the Cochrane library and EMBASE database. The search keywords included gastric cancer, stomach neoplasms, fat mass and obesity-associated gene, FTO, Fat mass and obesity associated gene, stomach neoplasms, gastric cancer, gastric neoplasms, stomach cancer, and stomach tumor. The search criteria were adapted to each specific database. The search period encompassed the inception of the databases up to May 2023. The search was limited to Chinese and English language publications, and relevant references were traced in the retrieved articles.

### 2.2. Inclusion and exclusion criteria

#### 2.2.1. Inclusion criteria.

Case-control studies or prospective/retrospective clinical studies. Patients diagnosed with gastric cancer based on pathological diagnosis. Studies investigating the expression of FTO and its correlation with clinical pathological indicators and prognosis in gastric cancer patients. Detection of FTO expression in gastric cancer samples using immunohistochemistry (IHC) or quantitative reverse transcription-polymerase chain reaction (qRT-PCR). Studies reporting data on the correlation between FTO expression levels and clinical pathological information as well as prognosis in gastric cancer patients.

#### 2.2.2. Exclusion criteria.

Studies unrelated to gastric cancer or FTO. Non-original articles, such as reviews, conference abstracts, letters, case reports, or meta-analyses. Studies lacking clinical pathological data or limited to cell-based assays. Duplicate data from the same study population.

### 2.3. Literature selection

Two researchers initially screened the literature by reading the titles and abstracts. Full texts were obtained for further exclusion or inclusion. Basic study information (first author’s name, publication year, country of study, and study population), experimental methods, characteristics of the participants (gender), and clinical parameters (differentiation type, depth of infiltration, lymph node metastasis, distant metastasis, TNM staging) were extracted from each study. The study’s prognostic outcomes, including overall survival (OS) and recurrence-free survival (DFS), were recorded. In case of any discrepancies, the literature was discussed among the research team or a third-party researcher was involved for judgment.

### 2.4. Assessment of literature quality

Two researchers used the Newcastle-Ottawa Scale (NOS) to assess the quality of each study. The NOS scoring system was applied to evaluate each study according to the NOS scoring criteria. Studies with an NOS score of 7 or higher were considered to be of higher quality and eligible for inclusion in this meta-analysis.

### 2.5. Statistical methods

The combined effect size was evaluated using odds ratio (OR) and 95% confidence interval (CI) as the measure of association. The odds ratio (OR) was used as a relative risk in case-control retrospective studies to quantify the association between high FTO expression and clinical parameters of gastric cancer. Heterogeneity among the included studies was assessed using the chi-square test, and *I*^2^ values were used to determine the degree of heterogeneity. An *I*^2^ value less than 25% indicated mild heterogeneity, *I*^2^ values between 25% and 50% indicated moderate heterogeneity, and *I*^2^ values greater than 50% indicated substantial heterogeneity. If the *P* value was greater than or equal to 0.1 or *I*^2^ was less than or equal to 50%, indicating low heterogeneity, a fixed-effects model was used to calculate the pooled estimates. Conversely, a random-effects model was used. All statistical tests in this meta-analysis were conducted using Stata 12.0 software. A *P* value less than .05 was considered statistically significant.

## 3. Results

### 3.1. Literature screening results and process

**Figure 1.**
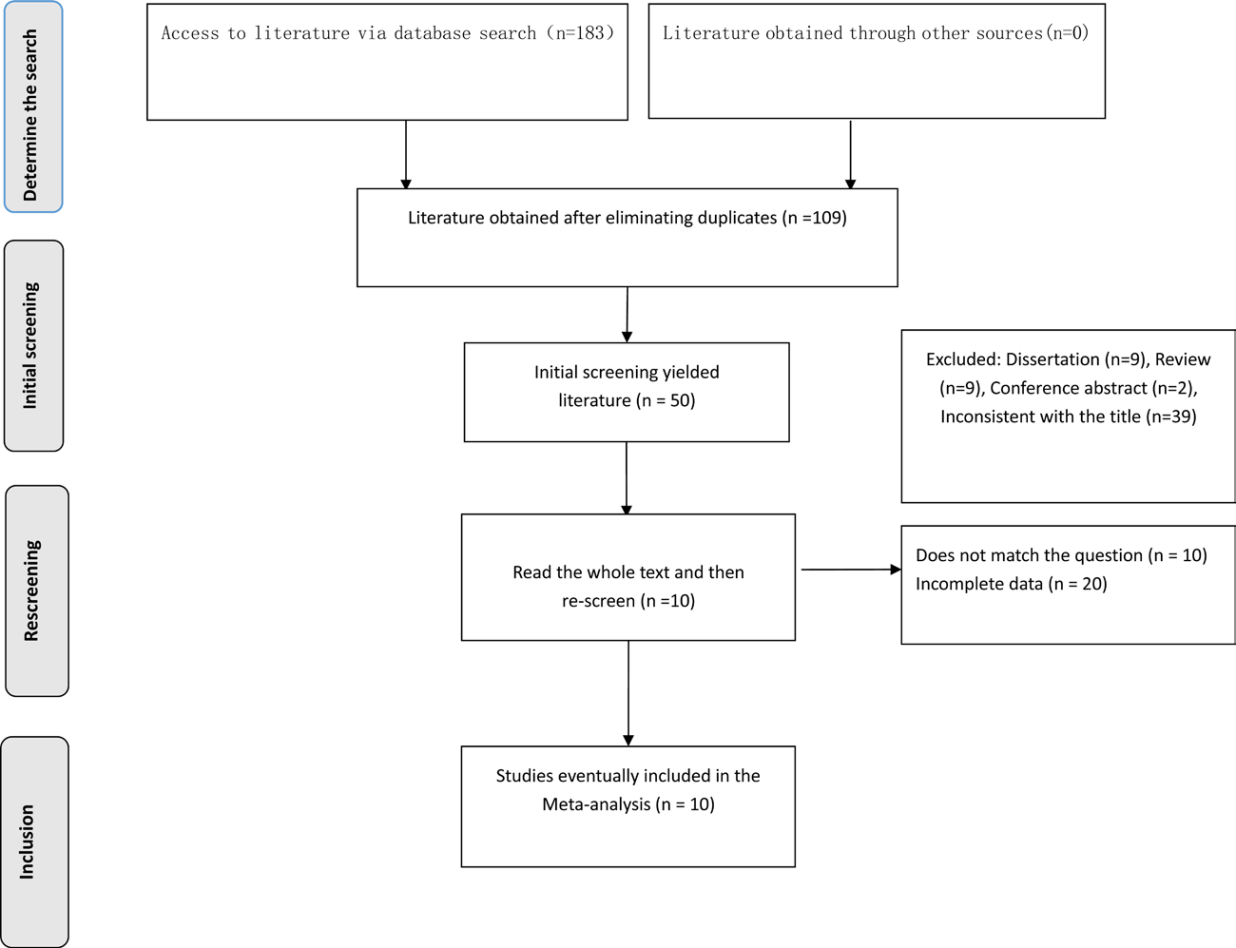
Flow chart of literature screening.

A total of 183 articles were obtained through the database search. After removing duplicate articles and reviewing titles, abstracts, and full texts, 10 articles were included. The detailed process of literature screening is presented in Figure 1.

### 3.2. Characteristics of included studies and quality assessment

According to the inclusion and exclusion criteria, a total of 10 retrospective studies were included in this study. These studies involved 1619 gastric cancer patients, and the samples used were tissue specimens. Immunohistochemistry was the main method used for FTO expression detection. The quality of the included studies was assessed using the NOS, with a maximum score of 9. All the included studies in this study scored ≥ 7. The basic characteristics of the included studies are summarized in Table 1.

**Table 1:** The characteristics of included studies.

Study	Country	Sample type	Test method	Sample size (n)	Gender M/F	Pathological indicators	Endpoints	NOS
Shimura T et al 2021	USA	FFPET	qRT-PCR	172	115/57	①②③④	NR	7
Xu D et al 2017	China	FFPET	IHC	128	68/60	①②⑤	OS	8
Yang X et al 2022	China	FFPET	IHC	87	68/19	①②③⑤	OS DFS	8
Liu C et al 2020	China	FFPET	IHC	102	60/42	①②③⑤	OS	8
Yang Z et al 2020	China	FFPET	IHC	64	35/29	①②⑤	NR	8
Li Y et al 2019	China	FFPET	IHC	450	208/142	①②⑤	OS PFS	7
Gui S et al 2023	China	FFPET	IHC	378	242/136	①②	NR	8
Guan K et al 2020	China	FFPET	IHC	20	4 ∕ 16	NR	OS RFS	7
Zhang J et al 2020	China	FFPET	IHC/qRT-PCR	128	82/46	NR	OS DFS	7
Zhou Y et al 2022	China	FFPET	qRT-PCR	90	66/24	①②④	NR	7

DFS = disease-free survival, F = female, FFPET = formalin-fixed, paraffin-embedded tissues, IHC = Immunohistochemistry, M = male, NOS = the Newcastle–Ottawa scale, NR = not reported, OS = overall survival, PFS = progression-free survival, RFS = recurrence-free survival, RT-qPCR = reverse transcription-quantitative polymerase chain reaction.

① age; ② TMN stage; ③ Venous invasion; ④ Hepatic metastasis; ⑤ Differentiation.

### 3.3. Meta-analysis results

#### 3.3.1. Association between FTO expression and gender and age of gastric cancer patients.

**Figure 2.**
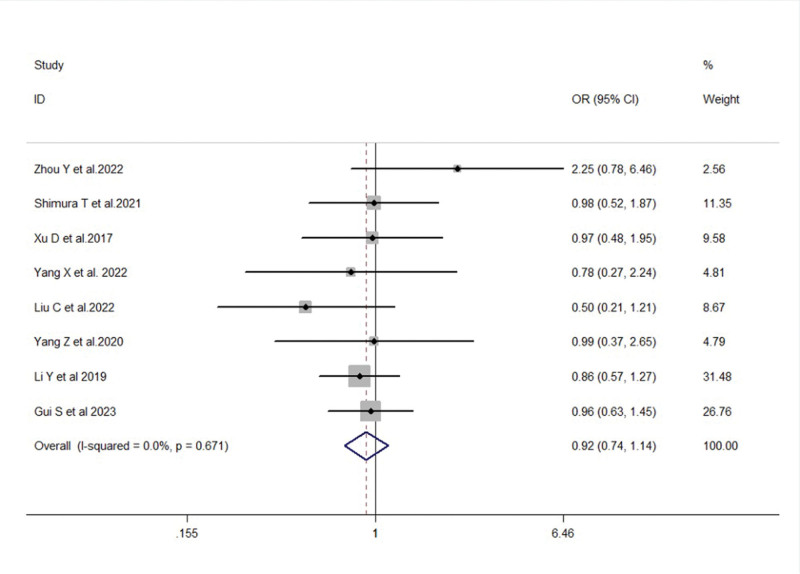
Forest plot of the correlation between FTO expression and gender of gastric cancer patients. FTO = fat mass and obesity-associated gene.

In terms of gender, a total of 8 studies reported the association between FTO expression and gender in gastric cancer patients. There was no significant statistical heterogeneity among the studies (*I*^2^ = 0.0%, *P* = .671). A fixed-effects model was used for meta-analysis, and the results showed no significant correlation between FTO expression and gender in gastric cancer patients (OR = 0.92, 95% CI: 0.74–1.14, *P* = .432) (Fig. 2).

**Figure 3.**
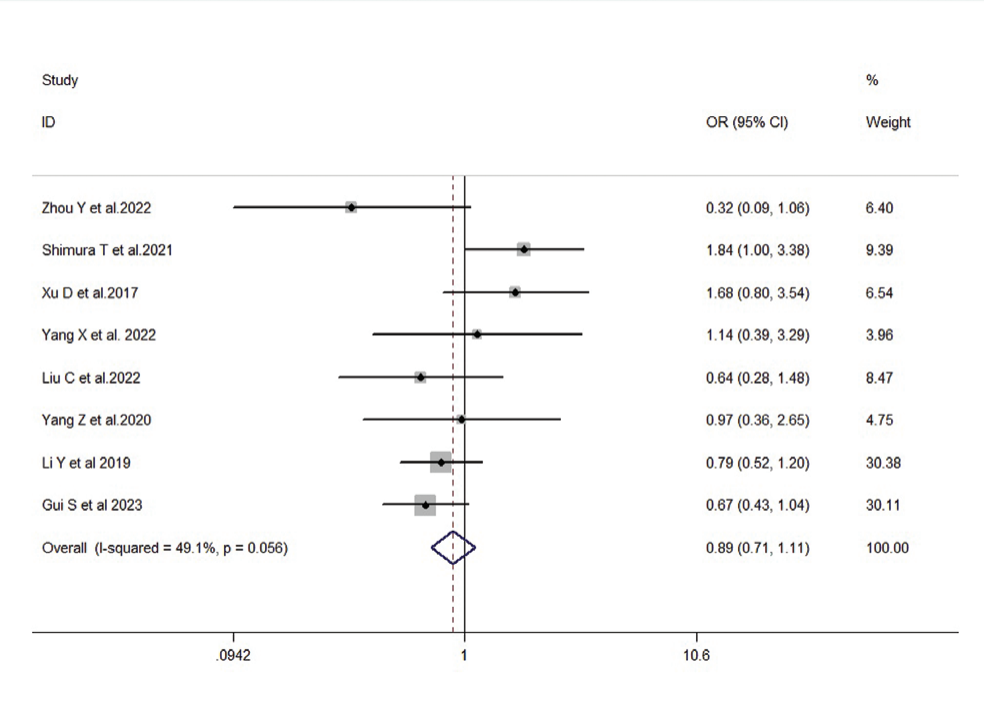
Forest plot illustrating the association between FTO expression and age in gastric cancer patients. FTO = fat mass and obesity-associated gene.

Regarding age, a total of 8 studies reported the association between FTO expression and age in gastric cancer patients. There was no significant statistical heterogeneity among the studies (*I*^2^ = 49.1%, *P* = .056). A fixed-effects model was used for meta-analysis, and the results showed that FTO expression levels in gastric cancer patients were not statistically significant in relation to age (OR = 0.89, 95% CI: 0.71–1.11, *P* = .306) (Fig. 3).

#### 3.3.2. Association between FTO expression and TNM staging and subgroup staging in gastric cancer patients.

**Figure 4.**
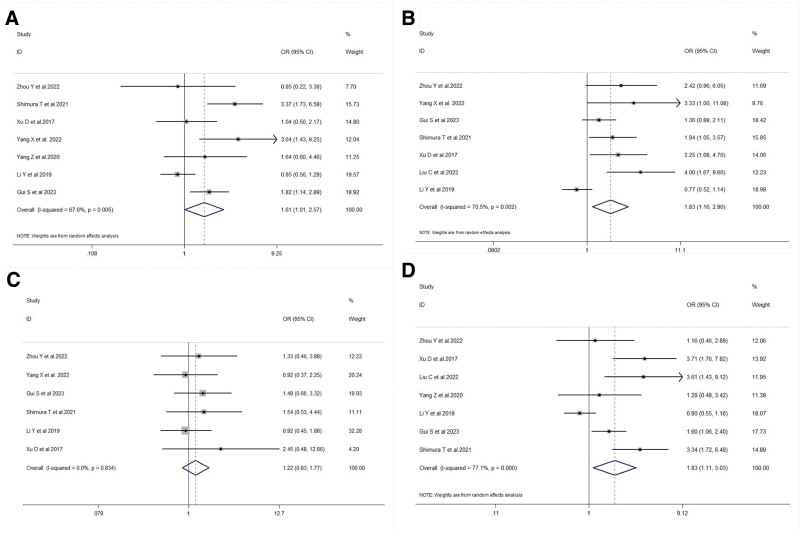
A: Forest plot illustrating the association between FTO expression and T staging in gastric cancer patients. B: Forest plot illustrating the association between FTO expression and N staging in gastric cancer patients. C: Forest plot illustrating the association between FTO expression and M staging in gastric cancer patients. D: Forest plot illustrating the association between FTO expression and TNM staging in gastric cancer patients. FTO = fat mass and obesity-associated gene, TNM = tumor node metastasis.

Regarding T staging, FTO overexpression was significantly associated with advanced T3 + T4 stage in gastric cancer patients (OR = 1.61, 95% CI: 1.01–2.57, *P* = .045) (Fig. 4A). Additionally, FTO overexpression was significantly associated with N2 + N3 stage (OR = 1.83, 95% CI: 1.16–2.90, *P* = .010) (Fig. 4B). However, there was no significant association between FTO expression and M stage (OR = 1.22, 95% CI: 0.83–1.77, *P* = .311) (see Fig. 4C). In terms of TNM staging, FTO expression levels were significantly increased in stage III + IV patients and were closely associated with advanced stages (OR = 1.83, 95% CI: 1.11–3.03, *P* = .019) (Fig. 4D).

#### 3.3.3. Association between FTO expression and tumor differentiation in gastric cancer patients.

**Figure 5.**
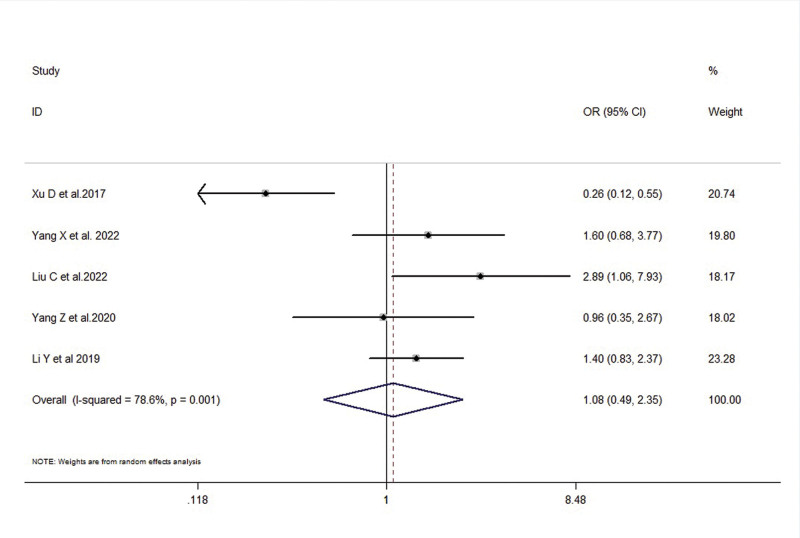
Forest plot illustrating the association between FTO expression and tumor differentiation in gastric cancer patients. FTO = fat mass and obesity-associated gene.

A total of 5 studies reported the association between FTO expression levels and tumor differentiation (poorly differentiated vs moderately to well-differentiated) in gastric cancer patients. There was a moderate level of heterogeneity among the included studies (*I*^2^ = 78.6%, *P* = .001), therefore a random-effects model was used. The meta-analysis results showed no significant correlation between FTO expression levels and tumor differentiation in gastric cancer patients (OR = 1.08, 95% CI: 0.49–2.35, *P* = .852) (Fig. 5).

#### 3.3.4. Association between FTO expression and liver metastasis in gastric cancer patients.

**Figure 6.**
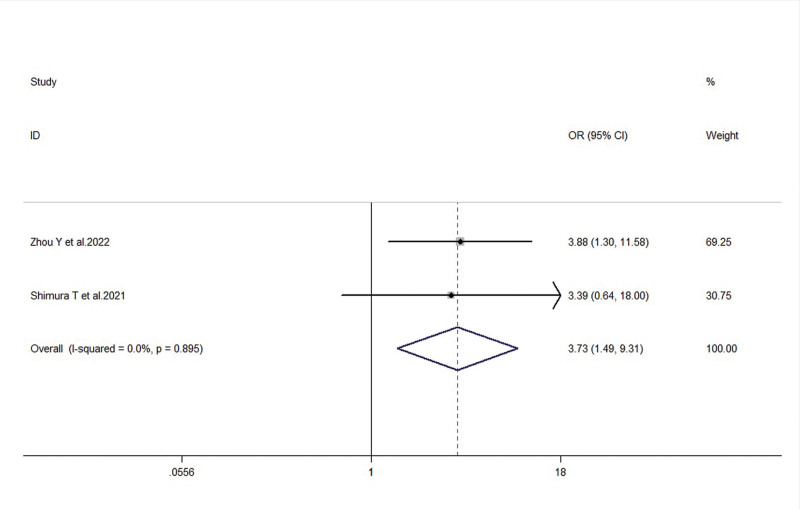
Forest plot illustrating the association between FTO expression and liver metastasis in gastric cancer patients. FTO = fat mass and obesity-associated gene.

Two studies reported the association between FTO expression levels and the presence or absence of liver metastasis in gastric cancer patients. There was low heterogeneity among the included studies (*I*^2^ = 0.0%, *P* = .895), therefore a fixed-effects model was used. The meta-analysis results showed that FTO expression levels were significantly higher in the liver metastasis group compared to the non-metastasis group, indicating a statistically significant difference (OR = 3.73, 95% CI: 1.49–9.31, *P* = .005) (Fig. 6).

#### 3.3.5. Association between FTO expression and vascular invasion in gastric cancer patients.

**Figure 7.**
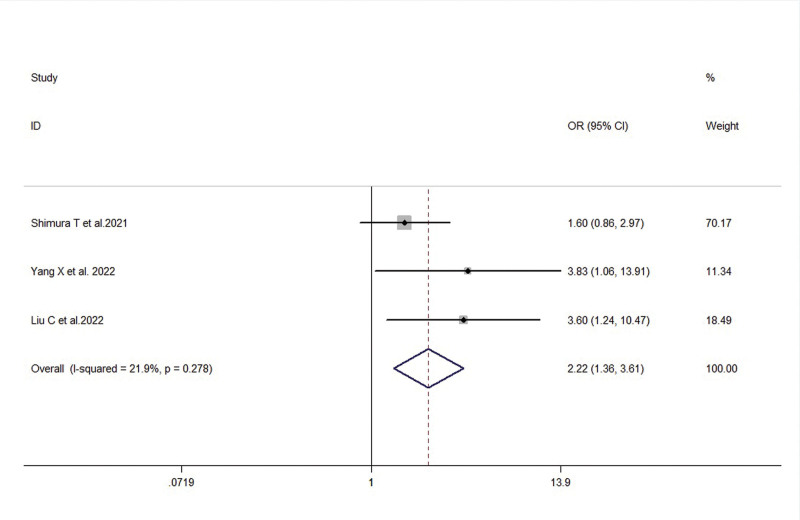
Forest plot illustrating the association between FTO expression and vascular invasion in gastric cancer patients. FTO = fat mass and obesity-associated gene.

Three studies reported the association between FTO expression levels and the presence or absence of vascular invasion in gastric cancer patients. There was low heterogeneity among the included studies (*I*^2^ = 21.9%, *P* = .278), therefore a fixed-effects model was used. The meta-analysis results showed that FTO expression levels were significantly higher in the vascular invasion group compared to the non-invasion group, indicating a statistically significant difference (OR = 2.22, 95% CI: 1.36–3.61, *P* = .001) (Fig. 7).

#### 3.3.6. Association between FTO expression and prognosis in gastric cancer patients.

**Figure 8.**
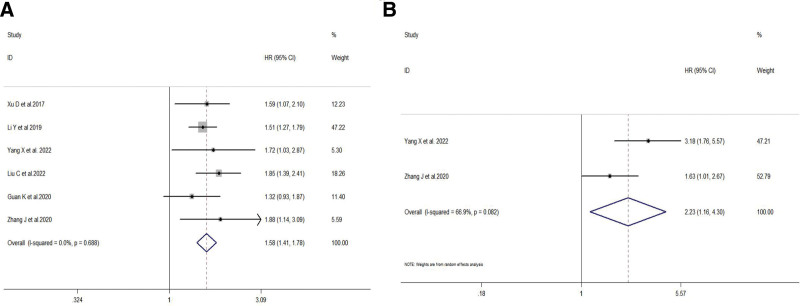
Association between FTO Expression and Prognosis in Gastric Cancer Patients A: Forest plot illustrating the association between FTO expression and overall survival in gastric cancer patients. B: Forest plot illustrating the association between FTO expression and recurrence-free survival in gastric cancer patients. FTO = fat mass and obesity-associated gene.

Six studies investigated the association between FTO expression levels and OS in gastric cancer patients. There was low heterogeneity among the included studies (*I*^2^ = 0.0%, *P* = .688), therefore a fixed-effects model was used. The meta-analysis results demonstrated that high FTO expression was an independent risk factor for poor overall survival in gastric cancer patients (HR = 1.58, 95% CI: 1.41–1.78, *P* < .001) (Fig. 8A). Two studies investigated the association between FTO expression levels and recurrence-free survival (DFS) in gastric cancer patients. There was moderate heterogeneity among the included studies (*I*^2^ = 66.9%, *P* = .082), therefore a random-effects model was used. The meta-analysis results revealed a significant correlation between high FTO expression and poor RFS in gastric cancer patients (HR = 2.23, 95% CI: 1.16–4.3, *P* = .016) (Fig. 8B).

### 3.4. Publication bias and sensitivity analysis

**Figure 9.**
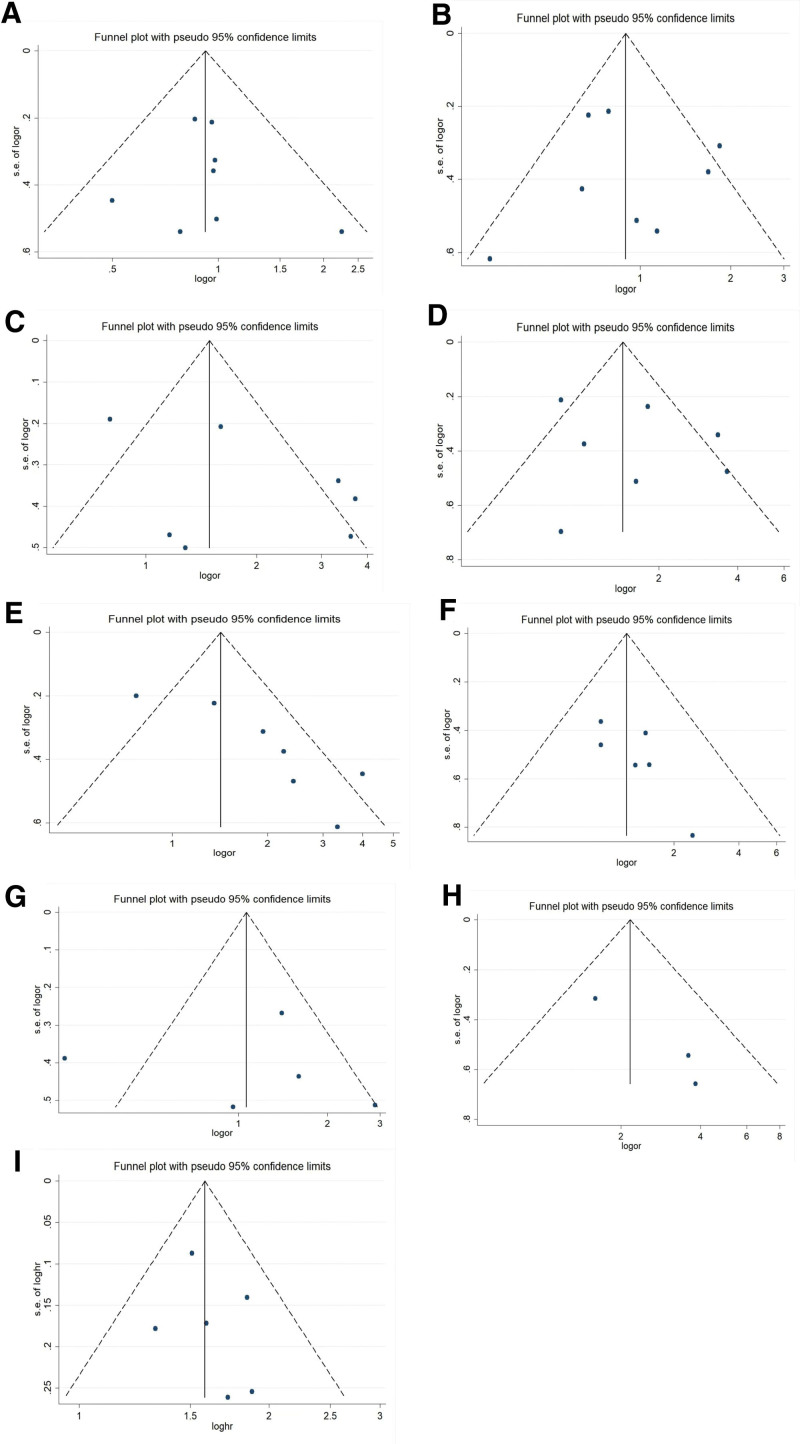
Funnel plot for publication bias test of FTO related studies. A, Gender; B, Age; C, T stage; D, N stage; E, M stage; F stage, TNM stage; G, Differentiation; H, Venous invasio; I, OS. FTO = fat mass and obesity-associated gene, TNM = tumor node metastasis.

In our meta-analysis, funnel plots as well as Egger’s tests were introduced to examine potential publication bias. A funnel plot of every group was conducted with OR/HR as the x-axis and stand error (SE) of log OR/HR as the y-axis, respectively. All of the plots were symmetric, indicating that publication bias was low (Fig. 9). In accordance with the results of funnel plots, little publication bias was identified by Egger’s tests (Table 2).

**Table 2:** Main results for meta-analysis between FTO and clinicopathological features/prognosis and publication bias (Egger’s test).

Correlation between FTO and clinicopathological features/ prognosis	No. of studies	Overall OR/HR (95% CI)	*P*_OR/HR_	Heterogeneity test (*I*^2^, *P*_bias_)	Publication bias (Egger’s test) (*t*, *P*_publication bias_)
Gender (male/female)	8	0.92 (0.74, 1.14)	0.432	0.0%, 0.671	0.35, 0.742
Age (≥60/<60)	8	0.89 (0.71, 1.11)	0.306	49.1%, 0.056	0.16, 0.875
TNM stage (III–IV/I–II)	7	1.83 (1.11, 3.03)	0.019	77.1%, 0.000	1.58, 0.176
T (T3T4/T1T2)	7	1.61 (1.01, 2.57)	0.045	67.6%, 0.005	0.72, 0.503
N (N1N2N3/N0)	7	1.83 (1.16, 2.9)	0.010	70.5%, 0.002	4.11, 0.009
M (M1/M0)	6	1.22 (0.83, 1.77)	0.311	0.0%, 0.834	2.18, 0.095
Differentiation (Poor/Well, Moderate)	5	1.08 (0.49, 2.35)	0.852	78.6%, 0.001	0.05, 0.966
Venous invasion (positive/negative)	3	2.22 (1.36, 3.61)	0.001	21.9%, 0.278	4.88, 0.129
OS	6	1.58 (1.41, 1.78)	0.000	0.0%, 0.688	0.75, 0.497

FTO = fat mass and obesity-associated gene, HR = hazard ratio, M = metastasis, N = node, OR = odds ratio, OS = overall survival, T = Tumor, TNM = tumor node metastasis.

Sensitivity analyses were conducted to evaluate whether individual studies influenced pooled ORs or HR by excluding one study by turns. The sensitivity analysis indicated that no study substantially influenced pooled OR/HR. This shifted effect measured of all studies and clinicopathological features/Prognosis slightly, but did not change the significance level for any outcome, which confirmed the stability of meta-analyses.

## 4. Discussion

FTO is the first discovered N6-methyladenosine (m6A) demethylase in eukaryotic cells,^[19]^ belonging to the non-heme Fe II/α-KG-dependent dioxygenase AlkB family of proteins, which also includes ABH1 to ABH8,^[10]^ and is widely expressed in various human tissues.^[20]^ FTO and the components of the m6A methyltransferase complex co-localize with splicing factors involved in mRNA processing, promoting the regulatory role of m6A in RNA processing.^[20,21]^ Imbalance of m6A has been implicated in the occurrence of various diseases, including cancer,^[22,23]^ and increasing evidence suggests its close association with cancer cell proliferation, cellular stress, metastasis, and immune response.^[24]^

Aberrant expression of FTO plays a significant role in the occurrence and progression of gastric cancer. Studies have shown that high expression of FTO in gastric cancer promotes cell proliferation, migration, invasion, and chemoresistance.^[25–27]^ The specific mechanisms by which FTO influences gastric cancer progression remain unclear and require further exploration. Based on published research, the mechanisms by which FTO is involved in gastric cancer development include: FTO promotes the demethylation of caveolin-1, CDKAL1, and mitochondrial dynamic metabolism through m6A modification or enhances gastric cancer cell proliferation, metastasis, and chemoresistance by reducing the methylation of integrin β1 (ITGB1)^[26–28]^; FTO also enhances PI3K/Akt signaling to promote gastric cancer cell proliferation and migration.^[29]^ These findings suggest that FTO can serve as an effective prognostic biomarker for gastric cancer. HDAC3 targets the FTO promoter region, specifically binding to FOXA2, inhibiting its transcriptional activity in gastric cancer cells, and thereby promoting cell proliferation, migration, and invasion.^[30]^ Upregulated FTO in gastric cancer cells promotes cisplatin resistance by regulating ULK1-mediated autophagy induction^[31]^; Omeprazole affects the chemosensitivity of gastric cancer cells through FTO-mediated upregulation of mTORC1 and DDIT3.^[32]^ FTO enhances the stemness of gastric cancer cells.^[33]^ Some studies have demonstrated the expression of FTO in gastric cancer and its correlation with clinicopathological features, but further research on prognosis outcomes is lacking.^[25,27,30]^

This study comprehensively analyzed the correlation between FTO expression and clinicopathological features of gastric cancer, including gender, age, and TNM staging, as well as its impact on survival outcomes. The results showed that FTO expression was higher in patients with advanced TNM stages (III-IV), deeper local invasion, lymph node metastasis, liver metastasis, and vascular invasion in gastric cancer. Moreover, high expression of FTO was significantly associated with poorer OS and recurrence-free survival (DFS) in gastric cancer patients (*P* < .001). The study also found that high FTO expression was correlated with shorter median progression-free survival (HR = 1.51, 95% CI: 1.42–2.24, *P* < .001) and recurrence-free survival (RFS) (HR = 1.47, 95% CI: 1.07–2.02, *P* < .001).^[34,35]^ Therefore, FTO may serve as a novel biomarker for predicting the prognosis of gastric cancer and as a potential target for therapeutic interventions.

## 5. Limitations

Although this study utilized a systematic meta-analysis approach, there are several limitations that should be acknowledged. Firstly, the number of included studies in this research is limited, which may affect the representativeness of the sample to some extent, there is significant heterogeneity in analysis of FTO and several clinicopathological features, thus random effects model was chosen to determine pooled ORs. Secondly, the inconsistency in the criteria used by different studies to determine FTO expression levels could impact the accuracy of the results. Thirdly, the study population mainly consisted of Asian individuals, and there may be variations in other racial and ethnic groups. Therefore, further confirmation of the conclusions of this study requires more high-quality, large-sample, multicenter randomized controlled trials.

## 6. Conclusions

In summary, high expression of FTO is closely associated with the pathological features of gastric cancer and affects the prognosis of gastric cancer patients. This study provides potential targets for the clinical diagnosis and prognosis of gastric cancer, However, in regard to the small number of studies included this meta-analysis, this standpoint needs further verification by incorporating more survival-related studies in future.

## Author contributions

**Data curation:** Ciba Zhu, Jichun Ma, Chenglou Zhu.

**Formal analysis:** Ziyao Wu, Xinqiao An, Dandan Ji.

**Resources:** Ciba Zhu, Chunling Xu, Jichun Ma, Chenglou Zhu.

**Investigation:** Ciba Zhu, Chunling Xu.

**Methodology:** Ciba Zhu.

**Project administration:** Chunling Xu.

**Writing – original draft:** Ciba Zhu, Chunling Xu.

**Writing – review & editing:** Ciba Zhu, Chunling Xu.
